# Attenuation of Infectious Bronchitis Virus in Eggs Results in Different Patterns of Genomic Variation across Multiple Replicates

**DOI:** 10.1128/JVI.00492-19

**Published:** 2019-06-28

**Authors:** Michael S. Oade, Sarah Keep, Graham L. Freimanis, Richard J. Orton, Paul Britton, John A. Hammond, Erica Bickerton

**Affiliations:** a The Pirbright Institute, Woking, United Kingdom; b University of Glasgow, Glasgow, United Kingdom; Loyola University Chicago

**Keywords:** attenuation, coronavirus, genomic variation, high-throughput sequencing, infectious bronchitis virus

## Abstract

The gammacoronavirus infectious bronchitis virus (IBV) causes an acute, highly contagious respiratory disease of poultry. Live attenuated vaccines are traditionally generated by serial passage of a virulent strain in embryonated chicken eggs; however, the molecular mechanism of attenuation is unknown. M41-CK, a virulent lab-adapted strain of IBV, was egg passaged over 100 times in four parallel independent replicates. All four final egg-passaged viruses were attenuated *in vivo* and exhibited similar growth phenotypes in adult chicken kidney cells and *ex vivo* tracheal organ cultures. The virus populations were sequenced by 454 pyrosequencing at the end of passaging, and the results showed that overall sequence diversity in the IBV population increased but the four replicates only had between 11 and 17 consensus-level single nucleotide polymorphisms (SNPs). Although hot spots of variation were identified in spike and nucleocapsid structural proteins as well as the 3ʹ untranslated region, each attenuated virus possessed a different pattern of genomic variation. Overall, only a small number of consensus-level SNPs were acquired during egg passage, leaving a potentially short route back to virulence. These results highlight the unpredictable nature of attenuation by serial egg passage and the need to develop mechanisms to rationally attenuate IBV for the next generation of effective vaccines.

**IMPORTANCE** Infectious bronchitis remains a major problem in the global poultry industry, despite the existence of many different vaccines. IBV vaccines are currently developed by serial passage of a virulent strain on embryonated hen’s eggs until attenuation; however, little is known about the evolution of the viral population during the process of attenuation. High-throughput sequencing of four replicates of a serially egg-passaged IBV revealed a different pattern of genomic variation in each attenuated replicate and few consensus-level SNPs. This raises concerns that only a small number of genomic mutations are required to revert to a virulent phenotype, which may result in vaccine breakdown in the field. The observed hot spots of variation in the attenuated viruses have the potential to be used in the rational attenuation of virulent IBV for next-generation vaccine design.

## Abstract (precis)

Infectious bronchitis remains a major problem in the global poultry industry, despite the existence of many different vaccines. IBV vaccines are currently developed by serial passage of a virulent strain on embryonated hen’s eggs until attenuation; however, little is known about the evolution of the viral population during the process of attenuation. High-throughput sequencing of four replicates of a serially egg-passaged IBV revealed a different pattern of genomic variation in each attenuated replicate and few consensus-level SNPs. This raises concerns that only a small number of genomic mutations are required to revert to a virulent phenotype, which may result in vaccine breakdown in the field. The observed hot spots of variation in the attenuated viruses have the potential to be used in the rational attenuation of virulent IBV for next-generation vaccine design.

## INTRODUCTION

Avian infectious bronchitis (IB) is a highly contagious respiratory disease of domestic fowl representing one of the most significant threats to poultry health worldwide. While a global problem, individual strains predominately remain unique to distinct geographical regions, with limited antigenic similarities between strains (1) and resulting in limited vaccine cross protection. Typically, morbidity is 100% for pathogenic strains of the causative agent, although mortality tends to be low. Despite this, some strains may cause nephritis or facilitate secondary bacterial infections, increasing mortality up to 50% (2). In addition, some serotypes demonstrate tropism for the oviduct, directly causing decreased egg quality and production (3). Poultry is an important food source worldwide, with an estimated 55 billion chickens produced worldwide per annum, including 5 billion for egg production. It has been estimated that every 10% reduction in IBV would be worth around £654 million to the global poultry industry (4).

The etiological agent of IB is infectious bronchitis virus (IBV), a gammacoronavirus belonging to the *Nidovirales* order, *Coronaviridae* family, species *Avian coronavirus*. The positive-sense single-stranded RNA viral genome is typically 27 to 28 kb and encodes the spike, envelope (E), membrane (M), nucleocapsid (N), and structural proteins and accessory proteins 3a, 3b, 4b, 5a, and 5b in the 3ʹ end of the genome. A large replicase gene is located at the 5ʹ end of the genome, which expresses two polyproteins, pp1a and pp1ab. The two polyproteins are cleaved by virus-encoded proteases, resulting in 15 nonstructural proteins (nsps); nsp2 to nsp11 are encoded by pp1a, and nsp2 to nsp10 and nsp12 to nsp16 are encoded by pp1ab, the latter produced as the result of a −1 ribosomal frameshifting mechanism. Untranslated regions (UTR) are located at the 5ʹ and 3ʹ ends of the genome. Unusually for an RNA virus, nsp14 exhibits 3ʹ-to-5ʹ exoribonuclease activity (5) which, in conjunction with nsp12, an RNA-dependent RNA polymerase (RdRp), forms part of a proofreading system that facilitates a high-fidelity replication of both genomic and subgenomic RNAs. The extent to which the proofreading capability of IBV impacts genetic variability, and the role of genetic diversity within an IB viral population, remains undefined despite having a discernible *in vivo* effect (6).

Although both live attenuated and inactivated vaccines are used as the primary control against IB, live attenuated virus vaccines offer easy application and greater immunogenicity/protection against disease (3). Risks of using live attenuated vaccines include breakdown of the vaccine or reversion to virulence (7, 8), and current European regulation requires such vaccines to include the demonstration of stability of attenuation over 6 *in vivo* passages. The process of generating live attenuated IBV vaccines typically involves serial passaging of wild-type isolates in embryonated specific-pathogen-free (SPF) eggs for up to 100 passages, resulting in a loss of pathogenicity in the final virus (9). Despite being a well-established method, the exact mechanisms underlying IBV attenuation have yet to be determined. The replicase (10, 11), structural (12), and accessory (13) genes have all been implicated in IBV pathogenicity.

Vaccine generation by egg passaging is likely to require a balance between a resulting loss of pathogenicity and maintaining immunogenicity. The process of attenuation by egg passage is not, however, guaranteed, as it is possible for viruses to remain virulent at the end of serial passaging. It is also unclear as to whether attenuation is linked to the adaptation of the virus to growth in the egg or another unknown mechanism.

It has been previously been suggested that the regulation of IBV evolution is the result of two factors: generation of genetic diversity and selection (14). Here, given the potential for an RNA virus to mutate, it is conceivable that *de novo* attenuating mutations occur and are selected for during egg passage. Alternatively, it is possible that given the highly diverse genetic structure of RNA virus population, the so-called “viral swarm,” low-frequency attenuated viruses could preexist within a virulent population, with the process of egg passaging driving selection of such attenuated genotypes. Additionally, recombination reported in the field will likely have a role in the evolution of IBV (15, 16). Regardless, understanding the diversity of an IBV intrahost population is critical to deciphering of the mechanisms of IBV attenuation.

Massively parallel sequencing technologies allow the intrahost diversity between clusters of variant viruses to be measured at great depth. Previous work involving next-generation sequencing and IBV was primarily focused on host immune responses (17) and characterization of actively circulating field strains (18–20). Conversely, the characterization of the genomic changes that confer attenuation has been extensively reported but is limited to Sanger sequencing (21, 22). In the present study, we serially egg passaged a lab-pathogenic strain of Massachusetts serotype, M41-CK, in four independent parallel replicates with the aim of generating four attenuated viruses. The resulting viruses have been characterized using *in vivo* and *in vitro* methods, confirming viral attenuation. We have deep sequenced these attenuated viruses to assess genomic differences both from the virulent parent and across multiple replicates of egg-passaged M41 and identified shared and unique sequence variation across these five viruses.

## RESULTS

### Generation of serially egg-passaged viruses.

A pathogenic strain of IBV belonging to the Massachusetts serotype lineage GI-1 (23), M41-CK, was used to inoculate four embryonated eggs to establish four separate virus lineages (A, B, C, and D), each starting from the same point of origin. After 13 subsequent passages, virus could no longer be detected in lineage B by routine reverse transcription-PCR (RT-PCR) screening (data not shown). At this point, lineage A was used to infect an additional egg to create an A1 lineage, replacing the original B passage, which remained independent for the remainder of the experiment. After a total of 106 egg passages (EP), pathogenicity was assessed for resultant EP106 viruses using *in vivo* and *in vitro* methods, with a further stock, EP107, grown solely for the purposes of 454 sequencing.

From this point, when lineages A to D are described, this refers to lineages A, A1, C, and D.

### Adaptation to embryonated eggs does not impair the ability of M41-CK to grow in adult chicken tissues *in vitro*.

**FIG 1:**
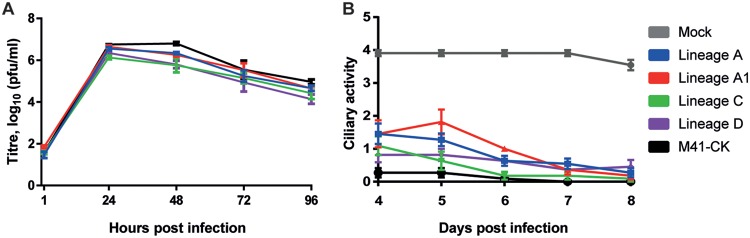
Serially egg-passaged viruses exhibit an *in vitro* phenotype similar to that of the virulent parent M41-CK. (A) Growth kinetics of serially egg passaged virus on CK cells. Confluent 6-well plates of CK cells were infected with virus at an MOI of 0.05. At 1, 24, 48, 72, and 96 h postinfection, cell medium was harvested for analysis of viral progeny by plaque titration assay on CK cells. Each value represents the mean of three replicates, with SEMs plotted as error bars. (B) Ciliary activity of viral growth in TOCs. TOCs were infected with 5 × 10^4^ PFU of IBV or mock infected with medium. Ciliary activity was assessed under a light microscope at days 4 to 8 postinfection and scored as follows: ≈100% activity = 4, ≈75% = 3, ≈50% = 2, ≈25% = 1, and ≈0% = 0. Each value plotted represents the mean score of 11 replicates, with SEMs plotted as error bars.

The growth characteristics of lineage A to D EP106 were investigated and compared to those of virulent M41-CK *in vitro* using primary chicken kidney (CK) cells. Viral progeny was assessed by plaque titration assay over a 96-h period (Fig. 1A). Growth characteristics were broadly similar for all five viruses, with lineages C and D at 48 h and lineage D at 96 h being significantly different from M41-CK (*P* = 0.0028, 0.0041, and 0.0222, respectively) using Dunnett’s multiple-comparison test. All other points showed no significant difference from M41-CK. Plaque morphologies for the egg-passaged viruses were similar to that of the M41-CK parent (data not shown).

To assess each virus’s ability to cause ciliostasis, tracheal organ cultures (TOCs) were infected with 5 × 10^4^ PFU of virus. At days 4 to 8 postinfection, ciliary activity was assessed by light microscopy and the proportion of beating cilia was determined (Fig. 1B). TOCs infected with virulent M41-CK exhibited very low ciliary activity and scored 0.27 and 0.09 for days 4 and 6 postinfection, respectively. Each serially passaged viral isolate caused a significant reduction in ciliary activity compared to that in the mock-infected group (*P* < 0.0001). Ciliary activities for lineage A (days 4 and 5), lineage A1 (days 4, 5, and 6), and lineage C (day 4) were deemed significant, with *P* values of <0.0001, 0.0004, <0.0001, <0.0001, 0.0015, and 0.0054, respectively. All other comparisons were nonsignificant (*P* > 0.05).

### Serially egg-passaged M41-CK viruses are attenuated *in vivo*.

**FIG 2:**
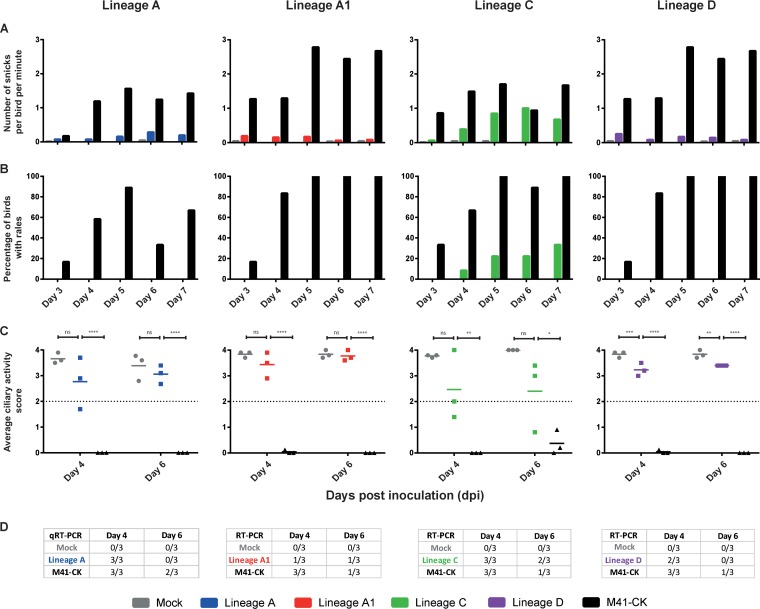
Serially egg-passaged M41-CK lineages are attenuated *in vivo*. Viral pathogenicity was assessed in a series of three separate animal studies. Assessment of lineages A and C was performed in separate investigations, while lineages A1 and D were assessed in a joint study. For each group (*n* = 12), 8-day-old SPF chicks were inoculated with 1 × 10^5^ PFU of each virus via the ocular and nasal routes. Clinical signs, including snicks per bird per minute (A) and rales (B), were recorded daily from days 3 to 7 postinfection. (C) Ciliary activity was assessed by observation of 10 tracheal cross sections (rings) from three randomly selected birds per group on days 4 and 6 postinfection. The mean ciliary activity of these three birds is plotted, with SEM plotted as error bars. *, *P* < 0.05; **, *P* < 0.01; ***, *P* < 0.001; ****, *P* < 0.0001. (D) Detection of IBV in trachea by using PCR or qPCR methods.

Groups of 12 8-day-old specific-pathogen-free (SPF) Rhode Island Red (RIR) chickens were inoculated with 1 × 10^5^ PFU of IBV or mock infected with 0.1 ml of serum-free medium via the intranasal and ocular routes. Each virus characterization had their own mock infection and virulent M41-CK virus controls. Snicking (Fig. 2A) and rales (Fig. 2B) were recorded daily between days 3 and 7 postinoculation.

Average ciliary activity scores over 50% suggest that IBV is attenuated, here indicated by an average ciliary activity score greater than 2 (Fig. 2C). On days 4 and 6 postinfection, the average ciliary activity of chickens infected with all serially egg-passaged M41-CK viruses remained above a 50% threshold and was significantly different from that of the M41-CK virulent controls. As RIR chickens are outbred, some differences were observed in clinical signs between the different M41-CK virulent control-infected groups; however, all virulent M41-CK-infected birds exhibited ciliostasis. With the exception of lineage D (*P* values of 0.0003 and 0.0044 for days 4 and 6, respectively), the difference between the egg-passaged viruses versus mock at each time point was deemed not significant by pairwise comparison (Tukey).

Chickens inoculated with lineage C demonstrated clinical signs consistent with IBV infection, although differences from mock-infected birds were not significant. While birds infected with lineage C showed some clinical signs, the virus was deemed attenuated due to low observed clinical effect and an average ciliary activity above 50%.

Tracheas were screened for the presence of IBV RNA on days 4 and 6 postinfection (Fig. 2D). IBV RNA was detected in the tracheas of all birds infected with virulent M41-CK on day 4 postinfection, decreasing to one or two out of three M41-CK-infected birds on day 6 postinfection. Similarly, the number of birds infected with egg-passaged viruses testing positive for IBV RNA declined from day 4 to day 6 postinfection in all groups except lineage A1, for which only a single bird tested positive on each day. Interestingly, presence of IBV RNA in the trachea and reduction in ciliary activity did not correlate. For example, while the average ciliary activities for birds infected with lineage A did not differ between day 4 and day 6 postinfection (Fig. 2C), all three birds infected with lineage A tested positive for IBV RNA on day 4 postinfection and none of the birds tested positive for IBV RNA in the trachea on day 6 postinfection (Fig. 2D).

Due to the reduction in clinical signs, reduction in the presence of IBV RNA in the trachea, and increased ciliary activity in birds infected with egg-passaged viruses in comparison to those of birds infected with virulent M41-CK, all four lineages were considered attenuated by serial egg passage.

### There are very few consensus-level sequence differences between virulent and attenuated M41-CK.

**FIG 3:**
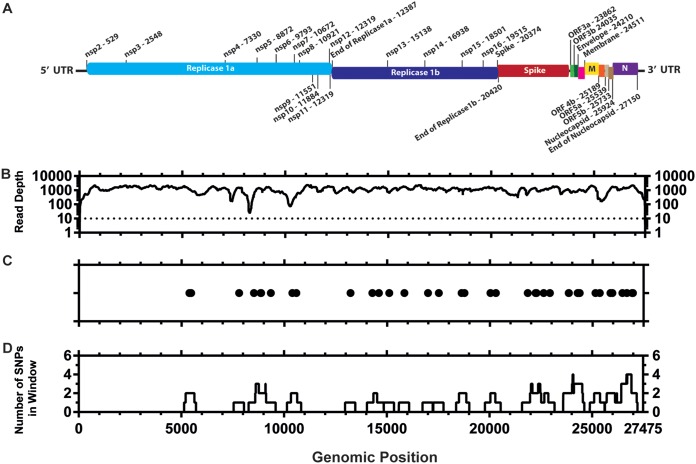
Distribution of consensus-level mutations across the M41-CK genome. (A) Genetic map of M41-CK consensus sequence (GenBank accession number MK728875). The nucleotide positions of each genomic region are detailed. (B) Sequencing coverage of aligned M41-CK quality-filtered reads to AY8512951.1 genome. Sites with no coverage (=13) have been plotted as having a coverage of 1. The dotted line shows site where consensus has not been called. (C) Consensus-level changes observed in M41-CK relative to AY8512951.1. Each circle represents a nucleotide substitution. A total of 39 mutations were identified between the two sequences. Consensus for M41-CK at the initial 18 positions at the 5ʹ end of the genome and final two positions at the 3ʹ end genome could not be called. (D) Number of consensus mutations occurring within a 499-nucleotide window with genomic position as the midpoint.

Once attenuation of the egg-passaged viruses was confirmed, we investigated the genomic changes for each isolate compared to the starting inoculum. Stocks of virulent M41-CK and attenuated EP106 viruses of each lineage were grown and partially purified, and the RNA genomes were sequenced by 454 pyrosequencing. Of the 105,728 quality-filtered reads obtained for M41-CK (Table 1), 102,415 (96.87%) reads aligned to M41 (GenBank accession number AY851295.1) (Fig. 3A and B). Thirty-nine point mutations (39/27,475) were identified in the M41-CK sequence relative to the sequence with accession number AY851295.1 (Fig. 3C and D), though the consensus for M41-CK could not be called for 20 nucleotide positions. This includes 13 positions that could not be called due to insufficient coverage and 7 positions that were ambiguous.

**TABLE 1:** Read statistics for each sequenced virus

Statistic	Value for virus				
	M41-CK	Lineage A	Lineage A1	Lineage C	Lineage D
No. of raw reads	114,483	23,617	25,395	69,831	120,410
No. of quality-filtered reads	105,728	21,506	22,854	64,699	111,319
No. of quality-filtered AY851295.1-aligned reads	102,415				
No. of quality-filtered M41-CK EP5-aligned reads	102,417	21,078	21,521	63,253	108,522
Mean depth	1,185.97	264.71	272.47	735.83	1,226.48
Median depth	1,218	199	123	719	1,251
Range of depth^*a*^	Min, 0; max, 2,331	Min, 0; max, 1,331	Min, 0; max, 2,258	Min, 0; max, 1,971	Min, 0; max, 2,439

a Min, minimum; max, maximum.

**FIG 4:**
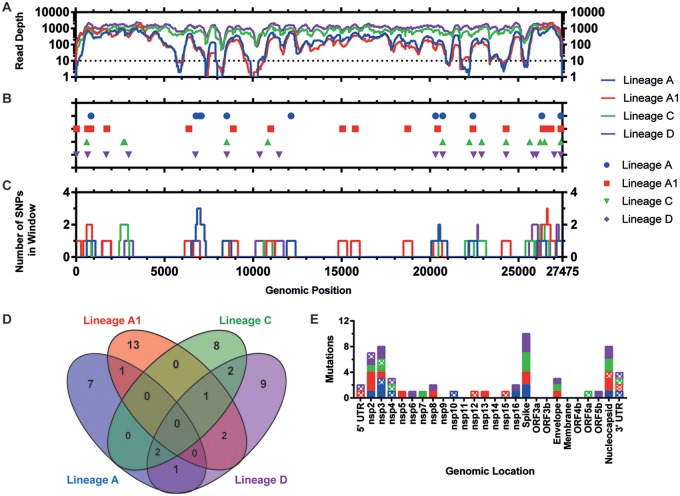
Distribution of consensus level mutations across attenuated egg-passaged virus genomes. (A) Sequencing coverage of aligned final egg-passaged virus reads to the M41-CK genome. The dotted line indicates positions where consensus and minor variants have not been called. Sites with no coverage have been plotted as having a coverage of one. Totals of 529, 111, 13, and 11 sites in lineages A, A1, C, and D, respectively, had no coverage (B) Consensus-level mutations observed in the attenuated viruses relative to M41-CK EP5. Each point represents a nucleotide substitution; insertions/deletions have not been plotted. Totals of 11, 17, 13, and 17 consensus-level mutations were identified in lineages A, A1, C, and D, respectively. Consensus could not be called for 2,858, 2,959, 18, and 18 positions, respectively, due to the minimum coverage threshold (inclusive of sites with no coverage). (C) Number of consensus-level mutations occurring within a 499-nucleotide window, 249 bp on either side of the midpoint position. (D) Venn diagram of consensus-level mutations occurring at the same position in more than one virus. (E) Synonymous and nonsynonymous mutations for each virus, for each genomic location. Nonsynonymous mutations are shown as solid colors, and synonymous mutations are shown in a checkered pattern. Here the 5ʹ UTR is defined as the genomic sequence upstream of the start of nsp2, while the 3ʹ UTR is defined the genomic sequence downstream of the nucleocapsid gene. Mutations occurring in these untranslated regions have been plotted as synonymous mutations.

With the set coverage threshold (10 reads), consensus could be called for 89.53%, 89.16%, 99.90%, and 99.90% of the viral genome for lineages A to D, respectively (Fig. 4A and Table 1). Consensus-level changes (occurring at a >50% frequency) were called for each lineage relative to M41-CK, with 11, 17, 13, and 17 single nucleotide polymorphisms (SNPs) identified in lineages A to D, respectively (Fig. 4B). Sliding-window analysis showed that some regions of the genome, particularly within the replicase gene, did not contain any consensus-level SNPs and may indicate more conserved areas of the viral genome (Fig. 4C). Conversely, mutations accumulated at the 3ʹ end of the genome, within the N gene and 3ʹ UTR.

A total of 9 SNPs were identified in more than one sample (Fig. 4D). Lineages A, C, and D all shared two mutations occurring at positions 8520 (nsp4, synonymous [S]) and 20720 (spike, K116R). Lineages A1, C, and D all shared a mutation at U24297C (envelope, F30L), with consensus at this site unable to be called for lineage A (coverage = 7). Reads at this position for lineage A concurred with the mutation present in the other three egg-passaged viruses.

Overall across all four passages, 43 (70.5%) of the identified consensus SNPs were nonsynonymous (NS), 11 (18.0%) were synonymous, and 7 (11.5%) occurred in untranslated regions (Fig. 4E).

### Distribution of variant positions indicates shared regions of diversity between attenuated viruses.

**FIG 5:**
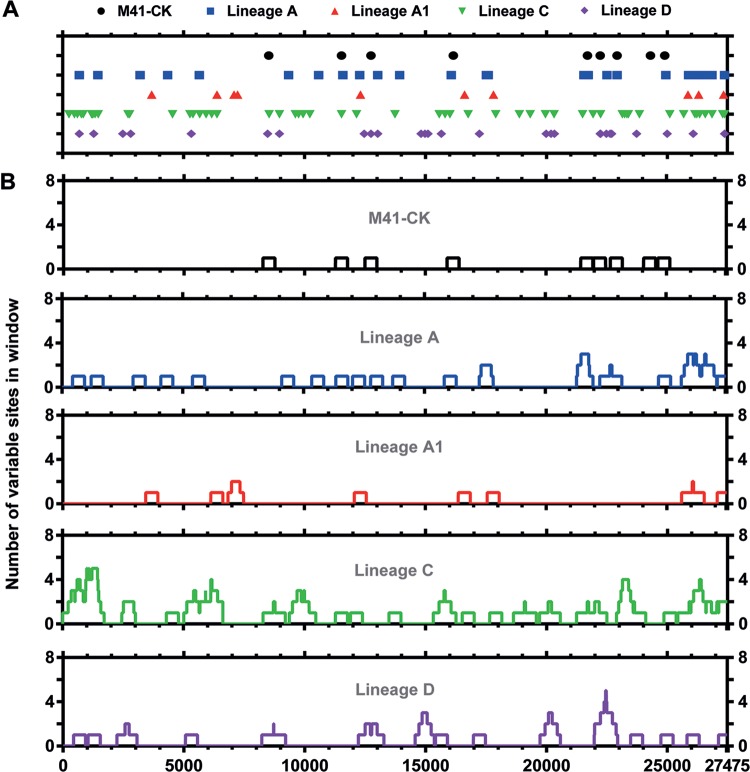
Final egg-passaged viruses possess greater variant density than virulent M41-CK. (A) Sites of variance identified relative to M41-CK consensus sequence. Totals of 9, 27, 10, 55, and 27 subconsensus variants were identified in M41-CK (relative to its own consensus), A, A1, C, and D, respectively. (B) Number of variants occurring within a 499-nucleotide window, 249 bp on either side of the midpoint (inclusive).

The location of subconsensus and consensus variant positions was determined and plotted across the length of the IBV genome (Fig. 5A; see also Table S1 in the supplemental material). In total, 156 unique polymorphisms were identified across each of the five lineages, with each exhibiting a diversity profile of variants unique to that lineage (Fig. 5B). This variant mapping suggests the emergence of new highly diverse regions in the attenuated virus rather than further diversification of preexisting regions present in the original population. Indeed, in most instances those variable regions present in the original population appear to have been lost in the final attenuated populations.

### Few consensus and subconsensus mutations are shared across attenuated viruses.

**FIG 6:**
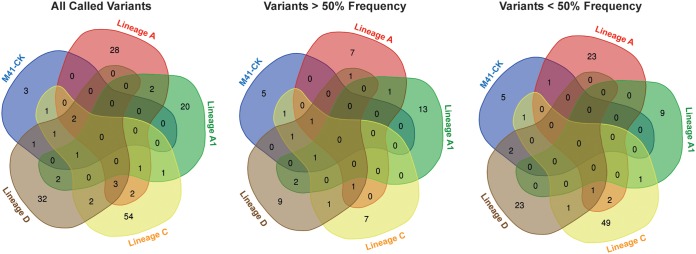
Final egg-passaged viruses have few shared consensus- and subconsensus-level variants. For each of the five viruses, three different lists of mutations were identified as follows: all mutations identified, mutations occurring at a frequency above 50%, and mutations occurring at a frequency below 50%. Each series of lists was then used to generate a Venn diagram using the Bioinformatics and Evolutionary genomics webtool. Numbers occurring in nonoverlapping regions are unique to that specific passage. No mutation at either consensus or subconsensus level was shared across multiple lineages.

Of those mutations identified, no mutation was common between M41-CK and all four attenuated viruses at either the consensus or subconsensus level. Indeed, no single mutation was shared in all four attenuated viruses. Twenty-three, 9, 49, and 23 subconsensus variants were identified in lineages A to D, respectively, that were unique to that one lineage, whereas 118 different subconsensus variants were identified across all five samples (Fig. 6).

All five consensus sequences were aligned to virulent M41 or to Ma5, H52, and H120 vaccine strain sequences available in the NCBI database. Only seven consensus-level changes resulting in a match to a vaccine strain sequence (i.e., Ma5, H52, or H120) were identified out of a total 153 SNPs within the egg-passaged viruses (Table 2). For U24297C other publicly available IBV sequences, both pathogenic and nonpathogenic, possess a C at this position, suggesting that this U is unique to our initial virus. Similarly, U22224 is present in both virulent M41 and vaccine strains Ma5, H52, and H120, indicating that mutation G22224U identified in lineage C is unlikely to be involved in attenuation.

**TABLE 2:**
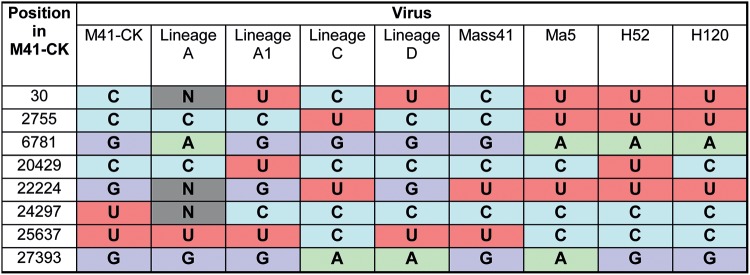
Comparison of identified consensus-level mutations against three vaccine strains of IBV^*a*^

a Generated consensus sequences for the five viruses were aligned to M41 (AY851295.1), Ma5 (KY626045.1), H52 (EU817497.1), and H120 (FJ881351.1) sequences available in the NCBI database using MAFFT. Instances where the nucleotide of at least one of the egg-attenuated viruses (i.e., lineages A to D) match a vaccine strain (i.e., Ma5, H52, and H120) were identified and are included here. M41-CK and M41 (AY851295.1) sequences were also included as a virulent M41 comparison. N, no consensus sequence at given position.

### No common trend in variant frequency is observed for mutations identified in virulent M41-CK.

Nine variants were detected within the original M41-CK population, three of which were no longer detectable in any egg-attenuated virus (Table 3). A11525G (nsp8, NS) occurs at the low initial frequency of 1.9% but only remained detectable in lineage C. Of the initial variants undetectable in the final serially egg-passaged populations, there was no apparent relationship between their initial and final frequencies; i.e., if a variant initially exists and remains within the population, it does not necessarily reach fixation. This capability for an intermediate frequency variant to exist within an attenuated population would suggest an intrinsic ability to retain a virulent genotype at a given position.

**TABLE 3:**
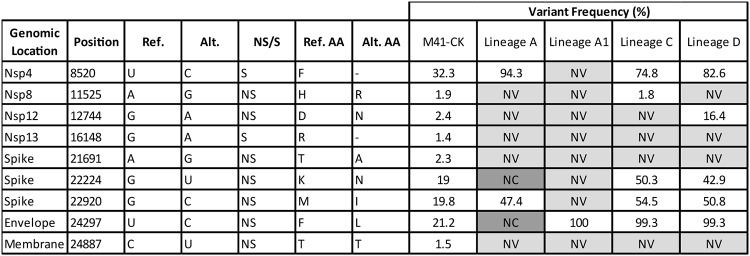
Resultant frequencies of minor variants detected in the original population^*a*^

a A total of nine sites of variance were identified in the original M41-CK population. Shown is the variance detected at these positions in the final viruses after serial egg passage. Positions where variants could not be detected are marked “NV” (no variance detected), whereas positions with insufficient coverage to call variants at are marked “NC” (no coverage). Genomic location, position, reference nucleotide, NS/S, and amino acid (AA) are relative to M41-CK consensus sequence. NS/S, nonsynonymous/synonymous variant. For NS variants, the resulting amino acid change is listed.

### Spike contains the highest number of subconsensus mutations; however, the 3ʹ UTR and N gene exhibit the highest rate of mutation.

**FIG 7:**
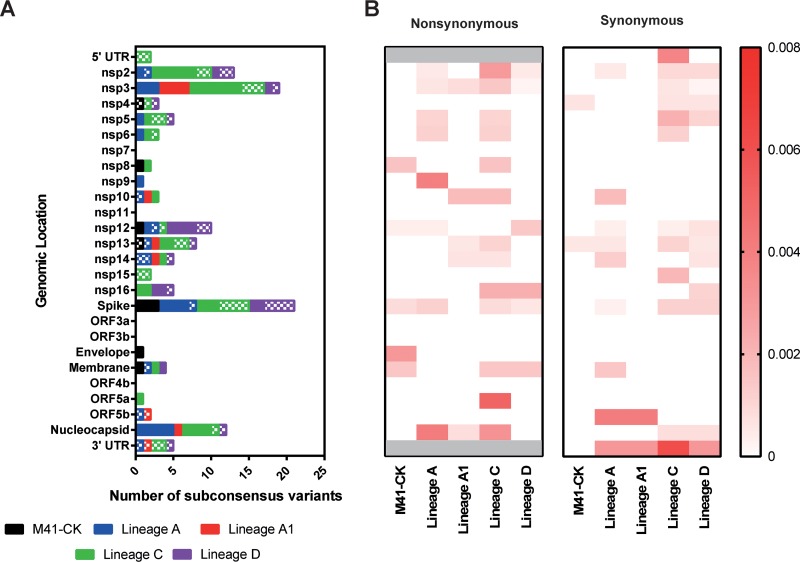
Numbers of nonsynonymous and synonymous subconsensus variants per genomic location and their rate of occurrence. Variants were called in each of the five viruses against the M41-CK consensus sequence. (A) Number of NS and S subconsensus mutations per genomic location. NS mutations are shown in solids, while S mutations are shown in a checkered pattern. Mutations occurring in the 5ʹ and 3ʹ UTR have been plotted as S mutations. (B) Count of NS/S variants was divided by the length of that genomic location to calculate the rate of variation per genomic location relative to length (base pairs). Variants occurring within the 5ʹ and 3ʹ UTR have been plotted as S mutations and have been shaded out on the NS heat map.

Each subconsensus variant was determined as possessing either an NS or S impact. The number of NS and S variants was quantified for each genomic location (Fig. 7A) relative to the coding gene length to provide a rate of substitution per nucleotide (Fig. 7B).

With the exception of lineage D, the majority of subconsensus variants in each lineage were NS. Spike, nsp3, nsp2, and nucleocapsid had the highest combined total of SNPs, with 21, 19, 13, and 12 SNPs, respectively. For spike, this consisted of 12 NS and 9 S mutations, the highest number of S mutations for a specific region. The highest number of NS mutations was observed in nsp3, with a total of 15. Combined totals of 2 and 5 mutations were identified in the 5′ and 3′ UTR, respectively. For the 3′ UTR these mutations did not occur in conserved stem-loop structure (24).

Relative to this length, the 3′ UTR had the highest rate of substitution, with an average of 0.003077 across all five passages (average = 0.003846 for attenuated viruses). Nucleocapsid exhibited the highest rate of NS change, with an average of 0.001630 (average = 0.002038 for attenuated viruses). Conversely, ORF5b possessed the highest rate of S change at 0.001626 (average = 0.002033 for attenuated viruses).

## DISCUSSION

Serial egg passaging of virulent IBVs to attenuate the virus has long been used as the standard procedure for generating a vaccine, with little to no understanding as to the mechanisms behind the attenuation. With the intention of furthering this field, we serially passaged M41-CK in four separate replicates to the point of attenuation and performed deep sequencing of the final-passage viruses to observe the resulting genomic differences and sequence variation. Our efforts identified few SNPs shared among all four of the attenuated final-passage viruses at both the consensus and subconsensus level. While one mutation (U24293C) was shared between all four attenuated viruses, C at the position is common between both pathogenic and nonpathogenic publicly available IBV sequences and is therefore unlikely to be involved in attenuation. This would imply that each attenuated isolate is attenuated by a different means (i.e., there is no single attenuating mutation shared across all four attenuated viruses).

Conversely, through our mutational analysis we have identified regions across the viral genome that undergo a high level of genetic change as the result of passaging and may contribute to attenuation. This has additionally identified areas of the genome where mutation does not occur and may not be evolutionarily tolerated, a feature which will equally assist future vaccine development.

Use of live attenuated viruses as vaccine candidates has long been supposed to have heightened risk of reversion to virulence (7, 25), despite the experimental demonstration of the stability of the clinical attenuation over some *in vivo* passages. While we could not ascertain the impact that each mutation has on pathogenicity in this study, our data demonstrate the possibility for the virus to maintain a virulent genotype within an attenuated population at a given position. This would indicate that the virus has the potential to selectively revert to virulence through selection of this subpopulation rather than the evolutionarily more challenging back-mutation to the original virulent genotype.

Compared to the starting population, each attenuated virus shows an apparent increase in sequence diversity at both the consensus and subconsensus levels after serial egg passaging. Due to the nature of serial passaging, it is possible that some observed variation occurred after the point at which these viruses would be deemed attenuated and suitable for vaccine use. Current strategies for characterizing the pathogenicity of IBV isolates largely rely on *in vivo* studies, as limited overlap is observed between *in vitro* and *in vivo* characteristics. Without a suitable alternative for assessing attenuation accurately, *in vivo* characterization remains the only representative means of assessing loss of pathogenicity. Given the number of samples involved here, it would not be ethical to perform pathogenicity experiments with each individual egg-passaged virus to establish the point at which our serial passages are deemed attenuated, as the harm would outweigh the benefit.

Recent advances in next-generation sequencing technologies provide the opportunity to sequence at a higher depth of coverage and the ability to identify ultralow-frequency mutations within a viral swarm. Surprisingly few variants within our virulent population were detected, with fewer still shared between the virulent and attenuated populations. This would first indicate that *de novo* mutation, as opposed to the selection of preexisting attenuating mutations (within the virulent population), is the cause of attenuation in this instance. Due to the coverage profile of our data set, the detection of diversity across the entirety of the genome, particularly in replicase and spike proteins of lineages A and A1, is limited. A consequence is that all mutations occurring in multiple lineages may not have been fully identified and detection of diversity in A and A1 is not wholly representative as a result. To fully answer this question of selection versus mutation, sequencing of intermediate passaging at a higher depth of coverage provided by current high-throughput sequencers would be required to show the change in frequency of variants at a higher temporal resolution. Such cross-sectional analysis would provide insight as to how the viral population evolves over the course of passaging.

While serial egg passaging does produce an attenuated virus, we have here demonstrated the inconsistent nature of this means of generating an attenuated IBV. From the same starting material, our data suggest that it is possible to differently attenuate four viruses with minimal overlap between these viruses. In addition, few of the SNPs identified in the attenuated viruses match the nucleotide found at these positions in commercial vaccine strains of the same serotype. This highlights that attenuation of IBV by serial egg passage results in different patterns of genomic variation each time. It is possible that the mutations identified here are linked by a means that have yet to be established. Regardless, the potential for industry to generate different vaccines against the same virulent strain with poor consistency is concerning. Moreover, the ability for potentially virulent subconsensus mutations to remain within an attenuated population demonstrates the risk of reverting to virulence and potentially has an evolutionary consequence when introduced into the field and in competition with other IBV strains. Regulations in place for market authorization in Europe do limit this risk. However, there is still an importance to move away from the process of attenuation by egg passage. Our findings would suggest a need for the genetic structure of live attenuated vaccine viruses generated by egg passaging to be fully characterized by deep sequencing, not only for purposes of quality control but also to improve our understanding of the mechanism of attenuation. This information, coupled with a reverse genetics system, would allow for the development of rationally attenuated IBVs with high efficacy and safety.

## MATERIALS AND METHODS

### Ethics statement.

All animal experimental protocols were performed in accordance with the UK Home Office guidelines and under license for experiments involving regulated procedures on animals protected under the UK Animals (Scientific Procedures) Act 1986. The experiments were performed in the Home Office licensed animal facilities of The Pirbright Institute, formerly the Institute for Animal Health (IAH), Compton, Newbury, United Kingdom, using specific-pathogen-free (SPF) Rhode Island Red (RIR) chickens and embryonated eggs obtained from the IAH Poultry Production Unit (PPU).

### Serial *in ovo* passaging.

A pathogenic strain of IBV belonging to the Massachusetts serotype lineage GI-1, M41-CK (23), at 2.6 × 10^4^ PFU in 100 μl of serum-free *N*,*N*-bis(2-hydroxyethyl)-2-aminoethanesulfonic acid (BES) medium, was inoculated into the allantoic cavities of four 10-day-old embryonated SPF RIR chicken eggs (A, B, C, and D). After a 24-h incubation, embryos were candled and culled by refrigeration for a minimum of 4 h. Allantoic fluid was then harvested, centrifuged at 700 × *g* for 5 min to clarify supernatant, and stored at −80°C. For subsequent passages, up to a total of 106, the allantoic fluid was diluted with BES medium between 1:100 and 1:10,000 based on visual inspection of the condition of the embryo and inoculated into the allantoic cavity, one egg per lineage, with each lineage remaining independent from each other.

Allantoic fluid was routinely screened for presence of IBV by RT-PCR amplification of the 3ʹ UTR, using oligonucleotides BG56 (5′-CAACAGCGCCCAAAGAAG-3ʹ) and 93/100 (5ʹ-GCTCTAACTCTATACTAGCCT-3ʹ). After routine screening at passage 14, lineage B could no longer be detected. At this passage allantoic fluid from lineage A was used to infect two eggs, A and A1, which remained independent for the duration of the experiment. The disappearance of lineage B could have been caused by multiple factors, both biological and procedural, and was not investigated any further, as it is known in the field that the loss of virus during egg passaging is not uncommon.

Where lineages A to D are described, this refers to lineages A, A1, C, and D.

### *In vitro* characterization using chicken kidney cells.

Chicken kidney (CK) cells were prepared from 2- to 3-week-old SPF RIR chickens (26). Confluent monolayers of CK cells in six-well plates were infected with viruses at a multiplicity of infection (MOI) of 0.05 in triplicate for each time point. Following attachment for 1 h at 37°C with 5% CO_2_, cells were washed with phosphate-buffered saline (PBS), 3 ml of BES-containing medium was added to each well, and cells were incubated at 37°C with 5% CO_2_. Supernatant was harvested at 1, 24, 48, 72, and 96 h postinfection and assayed in triplicate for progeny virus by plaque assay using CK cells. Two-way analysis of variance (ANOVA) followed by Dunnett’s multiple-comparison tests against the M41-CK virulent parent was performed using GraphPad Prism for Windows (version 8).

### *In vitro* characterization using tracheal organ cultures.

Chicken tracheal organ cultures (TOCs) were prepared from 19-day-old SPF RIR embryos (27). Groups of 11 TOCs were infected with 5 × 10^4^ PFU of IBV or mock infected with medium. Cultures were incubated for 1 h at 37°C in a rotating incubator, topped up with 500 μl of medium, and returned to a 37°C rotating incubator. The level of ciliary activity of each TOC was determined on days 4 to 8 postinoculation by light microscopy and scored as follows: ≈100% activity = 4, ≈75% = 3, ≈50% = 2, ≈25% = 1, and ≈0% = 0. The assessor was blinded to the experimental group. Two-way ANOVA followed by Dunnett’s multiple-comparison tests against the M41-CK virulent parent was performed using GraphPad Prism for Windows (version 8).

### *In vivo* analysis of the IBVs.

*In vivo* virus pathogenicity assessments were performed in a series of three separate experiments. Study of lineages A and C was performed in separate investigations, while A1 and D were assessed in a joint study. Groups of 12 8-day-old SPF RIR chicks were inoculated with 1 × 10^5^ PFU of virus or mock infected with PBS via the ocular and nasal routes. The chickens were housed in positive-pressure, HEPA-filtered isolation rooms, and each group was housed in a separate room.

### Assessment of pathogenicity.

The IBV-associated clinical signs used to determine pathogenicity were snicking, tracheal rales (a sound emanating from the bronchi, also detected by vibrations when holding a chick), and ciliary activity of the trachea. Chicks were observed daily between days 3 and 7 postinoculation. Snicks were counted by two persons over 2 min. Birds were checked individually for the presence of tracheal rales. Tracheas were removed from three randomly selected chickens from each group on days 4 and 6 postinfection for assessment of ciliary activity. Ten 1-mm sections were cut from three different regions of each trachea (three sections from the proximal, four from the middle, and three sections from the distal region). The level of ciliostasis of each tracheal section was determined by light microscopy; the assessor was blinded to the experimental group. Remaining birds were euthanized by a schedule one method on day 7 postinoculation and tissues harvested postmortem. A two-way ANOVA statistical test performed using GraphPad Prism for Windows (version 8) followed by Tukey pairwise comparison was used to determine significance.

### Detection of viral RNA.

Trachea stored in RNAlater was freeze-thawed and homogenized using the TissueLyser II (Qiagen) for 2 min at 25 Hz. Total RNA was isolated using an RNeasy minikit (Qiagen) by following the manufacturer’s animal tissue protocol with on-column DNase treatment.

The RNA isolated from tracheas extracted during the experiment to assess the pathogenicity of lineage A was screened for the presence of IBV using a Precision OneStep (PrimerDesign) quantitative RT-PCR (qRT-PCR) assay. No-template controls and positive controls provided in the kit were included in each qRT-PCR assay according to the manufacturer’s instructions. Samples were considered to contain IBV RNA if the threshold cycle (*C_T_*) value was greater than 17, the lower limit of detection.

cDNA was synthesized from RNA isolated from tracheas extracted during the experiments to assess the pathogenicity of lineages A1, C, and D using Superscript III reverse transcriptase (Invitrogen by Life Technologies) with a random oligonucleotide primer as per the manufacturer’s instructions. The cDNA was screened for the presence of IBV sequence by PCR using *Taq* DNA polymerase (Invitrogen by Life Technologies) and primers M20 (5ʹ-GGAATGGGCATAATAAGG-3ʹ) and M23 (5′-CACTGCTACCCCGTACCCG-3′) in nsp8 and nsp12, respectively. A positive control known to contain IBV RNA and a no-template negative control were used at the reverse transcription and PCR stages.

### Growth of viral stocks for sequencing.

As required for 454 pyrosequencing, stocks of each of the five viruses were grown in order to obtain sufficient purified RNA. To generate these stocks, 10 10-day-old embryonated SPF RIR chicken eggs per virus were infected with 100 μl of IBV-infected allantoic fluid (diluted 1:10,000 with BES medium) and incubated as detailed previously. Stocks of both the starting (M41-CK) and final (lineages A to D, passage 106) viral populations were grown for sequencing. Allantoic fluid from each viral group was pooled and centrifuged to clarify supernatant.

### Deep sequencing and sample preparation.

Partial purification of IBV and isolation of viral RNA for deep sequencing were performed as previously described (28). Briefly, IBV-infected allantoic fluid was purified by ultracentrifugation and the resulting virus pellet was resuspended in TRIzol reagent. RNA was extracted from supernatant as per the manufacturer’s instructions and quantified by NanoDrop assay. Samples were then sent to the Centre of Genomic Research (CGR), University of Liverpool, UK, for library preparation and sequencing. cDNA libraries were generated according to the *GS FLX Titanium cDNA Rapid Library Preparation Method Manual* (29) and sequenced using a GS FLX with Titanium Series and run protocol.

### Analysis of deep sequencing data and generation of a representative M41-CK consensus sequence.

Matching FASTA and QUAL files were consolidated to FASTQ format for each virus, and read quality was assessed using FastQC (30). Reads were quality trimmed using PRINSEQ v0.20.4 (31) with the following parameters: minimum length = 100, minimum quality mean = 25, maximum NS = 0, and trim quality right = 25. M41-CK quality-filtered reads were aligned to GenBank accession number AY851295.1 using bowtie2 v2.2.9 (32) with reads inputted as unpaired. All other settings were set as default. A consensus sequence was generated from this alignment where indel mutations were permitted and a minimum coverage of 10 reads was required for a nucleotide to be called (GenBank accession number MK728875). For positions where coverage was below this threshold, an ambiguity code, “N,” placed instead. This consensus sequence was annotated (Fig. 3A) according to information available (33, 34) and UniProtKB accession number P0C6Y3 (35), where regions of highly similar sequences were identified. The annotations of AY851295.1 available from NCBI were not used in this instance.

Quality-filtered reads for all five viruses were aligned to the M41-CK consensus sequence with bowtie2 (reads inputted as unpaired reads). As before, a consensus sequence for each of the final virus was generated with a minimum coverage threshold of 10. To ensure alignment to original sequence, indels were not permitted due to low confidence, as pyrosequencing is poor at resolving regions of low complexity. Minor variants were called for each virus using Lofreq* (version 2.1.2) (36) using default parameters. Variants were not called for positions where coverage was <10 reads. Comparison of shared mutations was performed using the Bioinformatics and Evolutionary Genomics webtool (http://bioinformatics.psb.ugent.be/webtools/Venn/) using position, reference nucleotide, and variant nucleotide as inputs.

### Data availability.

The sequence was deposited in GenBank under accession number MK728875.

## Supplementary Material

Supplemental file 1
